# De-escalation of P2Y12 Inhibitor Use After Percutaneous Coronary Intervention and Acute Coronary Syndromes

**DOI:** 10.1016/j.cjco.2021.04.010

**Published:** 2021-04-30

**Authors:** Quinton Barry, Angel Fu, Rene Boudreau, Alyssa Chow, Cole Clifford, Trevor Simard, Aun Yeong Chong, Alexander Dick, Michael Froeschl, Christopher Glover, Benjamin Hibbert, Marino Labinaz, Michel Le May, Juan Russo, Derek So, CAPITAL Investigators

**Affiliations:** Department of Cardiology, University of Ottawa Heart Institute, Ottawa, Ontario, Canada

## Abstract

**Background:**

De-escalation from potent platelet P2Y12 inhibitors to clopidogrel is common. Despite having a clinical rationale, non-bleeding-related de-escalation when a lateral change between potent agents is an option may put patients at increased ischemic risk. We set out to define the scope of P2Y12 inhibitor de-escalation in a large clinical registry and evaluate the potential impact of non-bleeding-related de-escalation on clinical outcomes.

**Methods:**

**:** A retrospective cohort study was performed on consecutive patients in the **Ca**rdiovascular **P**ercutaneous **I**ntervention **T**ri**al** (CAPITAL) registry to identify those who underwent a switch in therapy within 1 year of percutaneous coronary intervention. The de-escalations were categorized as bleeding-related or non-bleeding-related. The primary outcome was major adverse cardiovascular events, a composite of death, myocardial infarction, and stroke. Secondary outcomes included individual components of major adverse cardiovascular events and a safety endpoint of thrombolysis in myocardial infarction bleeding.

**Results:**

Of 1854 patients, 209 (11.3%) underwent de-escalation: 24.9% of cases were bleeding-related, 37.8% were non-bleeding-related, and 37.3% were for unknown reasons. All patients with non-bleeding-related de-escalation were switched from ticagrelor to clopidogrel. The primary outcome occurred in 14 (6.7%) patients, of which 50% underwent non-bleeding-related de-escalation (*P* = 0.430). Among those with non-bleeding-related de-escalation, 7.6% were hospitalized for myocardial infarction, compared to 1.9% and 3.8% among those with a bleeding-related and unknown rationale, respectively (*P* = 0.293).

**Conclusions:**

De-escalation, particularly non-bleeding-related de-escalation, of P2Y12 inhibitors is common. A substantial proportion of such de-escalation may be avoidable. Given the potential risk of ischemic complications, strategies should be considered to encourage both the upfront use of potent P2Y12 inhibitors and alternative strategies to de-escalation.

## Résumé

**Contexte:**

La désescalade thérapeutique consistant à passer d'un inhibiteur puissant du récepteur plaquettaire P2Y_12_ au clopidogrel est pratique courante. En dépit de son fondement clinique, la désescalade non liée aux saignements lorsqu'une substitution d'inhibiteurs puissants est possible peut entraîner une augmentation du risque d'ischémie chez les patients. L'objectif de notre étude était d'analyser, dans un vaste registre clinique, l'amplitude du recours à la désescalade à partir d'un inhibiteur du récepteur P2Y_12_ et d’évaluer les conséquences possibles de la désescalade non liée aux saignements sur les résultats cliniques.

**Méthodologie:**

Une étude de cohorte rétrospective a été effectuée sur une série de patients consécutifs inscrits au registre CAPITAL (***Ca****rdiovascular****P****ercutaneous****I****ntervention****T****ri****al***) afin de recenser ceux qui avaient fait l'objet d'un changement de traitement au cours de l'année suivant leur intervention coronarienne percutanée. Les désescalades ont été classées en deux catégories selon qu'elles étaient liées ou non liées aux saignements. Le critère d’évaluation principal, soit la survenue d'un événement cardiovasculaire indésirable majeur (ECIM), était un critère composite regroupant le décès, l'infarctus du myocarde et l'accident vasculaire cérébral. Les critères d’évaluation secondaires comprenaient chaque composante individuelle du critère composite et un critère d’évaluation de l'innocuité mesuré par le score TIMI (thrombolyse dans l'infarctus du myocarde) relatif aux saignements.

**Résultats:**

Sur 1854 patients, 209 (11,3 %) avaient fait l'objet d'une désescalade, qui était liée aux saignements dans 24,9 % des cas, non liée aux saignements dans 37,8 % des cas et sans raison indiquée dans 37,3 % des cas. Tous les patients ayant fait l'objet d'une désescalade non liée aux saignements étaient passés du ticagrélor au clopidogrel. Le critère d’évaluation principal a été observé chez 14 (6,7 %) patients, dont 50 % avaient fait l'objet d'une désescalade non liée aux saignements (*p* = 0,430). Parmi les patients ayant fait l'objet d'une désescalade non liée aux saignements, 7,6 % avaient été hospitalisés pour un infarctus du myocarde, comparativement à 1,9 % et 3,8 % des patients chez qui la désescalade était liée aux saignements ou n'avait pas de raison connue, respectivement (*p* = 0,293).

**Conclusions:**

La désescalade à partir d'inhibiteurs du récepteur P2Y_12_, et particulièrement la désescalade non liée aux saignements, est pratique courante, alors qu'elle pourrait être évitée dans une proportion élevée de cas. Compte tenu du risque de complications ischémiques d'une telle pratique, des stratégies devraient être envisagées afin d'encourager à la fois le recours dès le départ à des inhibiteurs puissants du récepteur P2Y_12_ et l'adoption de stratégies de remplacement de la désescalade.

Three oral platelet P2Y12 receptor inhibitors—clopidogrel, ticagrelor, and prasugrel—are available for use in conjunction with acetylsalicylic acid for dual antiplatelet therapy following acute coronary syndromes (ACSs) and percutaneous coronary intervention (PCI).^1^ Of the P2Y12 inhibitors, prasugrel and ticagrelor are more potent, with demonstrated ability to reduce the incidence of major adverse cardiovascular events (MACE), in comparison to clopidogrel.^2^, ^3^, ^4^ Thus, prasugrel and ticagrelor are recommended over clopidogrel in current guidelines as first-line agents in patients presenting with an ACS and undergoing PCI.^1^^,^^5^ Increased potency of these first-line P2Y12 inhibitors, along with differences in their mechanisms of action, can result in adverse drugs events or side effects. Accordingly, a change (i.e., a switch to the alternative first-line agent) or de-escalation of a P2Y12 inhibitor is not uncommon in clinical practice.^1^^,^^6^, ^7^, ^8^ Although evidence suggests that de-escalation should be avoided, sometimes it is justifiable to counterbalance bleeding risk.^1^ Conversely, when there is an alternative, de-escalation may deprive patients of the ischemic benefits of a more potent medication.

Recently, the medication with the brand name prasugrel was discontinued in Canada by its distributor, with the purported rationale being that the discontinuation was a business decision. We hypothesize that this change is the result of prasugrel underutilization in Canada, especially in the role as the drug of choice if discontinuation of ticagrelor is required.

There is a paucity of granular data identifying rationales for de-escalation in a real-world setting. Accordingly, we sought to quantify the rate of and indication for de-escalation of P2Y12 inhibitor therapy in a large contemporary registry, to further understand the relative underutilization of guideline-supported and best evidence–based potent antiplatelet therapy in Canada. We further set out to determine if the de-escalation could be avoided, with the ultimate aim of identifying patients who may derive benefit from a lateral change in therapy, in lieu of de-escalation.

## Materials and Methods

### Patient selection

This study was reviewed and approved by the Ottawa Health Science Network Human Research Ethics Board. This was a retrospective cohort study with prospective telephone follow up. Consecutive patients who underwent PCI between August 1, 2015 and December 31, 2016 were identified from the **Ca**rdiovascular **P**ercutaneous **I**ntervention **T**ri**al** (CAPITAL) registry at the University of Ottawa Heart Institute, which is a tertiary care center located in Ottawa, Canada with a catchment area of approximately 1.3 million people inclusive of 21 referral hospitals. Patients were included if they were over 18 years of age, underwent stent implantation during PCI, were discharged on a P2Y12 inhibitor, and had a switch of their P2Y12 inhibitor within 1 year of index PCI. Patients who did not survive the index procedure were excluded from this study. The choice of P2Y12 inhibitor was at the discretion of the treating physician.

### Data collection

The CAPITAL PCI database was used to compare patients’ prescribed P2Y12 inhibitors at the time of index PCI to that at 1-year follow-up, thus identifying patients who underwent de-escalation. Patients without documented follow-up at 1 year after their index PCI received a scripted telephone interview. In patients who underwent de-escalation, the rationale was investigated. If a rationale was not documented at the time of the switch in therapy, a comprehensive chart review of the patient's electronic medical record was performed. Specifically, all available documentation was reviewed, focusing on, but not limited to, cardiology, cardiac surgery, general internal medicine, and emergency medicine. If a rationale for de-escalation remained unidentified following the above chart review, the reasoning for de-escalation was labeled as unknown. Documentation outside of the electronic medical record from patients’ community cardiologists or family physicians was not sought out. In addition to the reason for switching the P2Y12 inhibitor, baseline demographic characteristics, indication for PCI, periprocedural characteristics, and medications including P2Y12 inhibitors were recorded pre-PCI, post-PCI, at discharge, and at 1-year follow up.

Switches of P2Y12 inhibitor were classified per the “2017 International Expert Consensus on Switching Platelet P2Y12 Receptor Inhibiting Therapies”^6^ as: (i) de-escalation with a switch from a more potent P2Y12 inhibitor (ticagrelor or prasugrel) to clopidogrel; (ii) escalation with a switch from clopidogrel to ticagrelor or prasugrel; or (iii) a switch between ticagrelor and prasugrel. For patients who underwent de-escalation, the de-escalation was further categorized as bleeding-related or non-bleeding-related, based on the rationale for de-escalation. Our definition of bleeding-related de-escalation was based on the recommendations in the “2018 Canadian Cardiovascular Society/Canadian Association of Interventional Cardiology Focused Update of the Guidelines for the Use of Antiplatelet Therapy”*.*^1^ De-escalation was deemed to be bleeding-related if it was for bleeding, need for concurrent oral anti-coagulation, intolerable side effects of or absolute contraindications to both potent P2Y12 inhibitors, or if PCI was conducted for a non-ACS indication. Absolute contraindications to prasugrel were defined as hypersensitivity to prasugrel, active bleeding, and prior transient ischemic attack or stroke.^9^ Absolute contraindications to ticagrelor were defined as hypersensitivity to ticagrelor, active bleeding, and history of intracranial hemorrhage. All other reasons were deemed to be non-bleeding-related. A third category of “unknown” was created for patients whose indication for de-escalation was not documented.

### Outcomes

The primary outcome was MACE, defined as a composite of death, myocardial infarction, and stroke. Only outcomes that occurred following de-escalation were counted towards the primary outcome. Secondary outcomes included individual components of MACE and a safety endpoint of bleeding classified using the thrombolysis in myocardial infarction (TIMI) bleeding classification. If bleeding was the indication for de-escalation, only subsequent bleeding events following de-escalation were counted towards the secondary outcome.

### Statistical methods

Patients with bleeding-related reasons for de-escalation were compared to those with non-bleeding-related and unknown reasons for de-escalation. For comparison of continuous variables across all 3 de-escalation groups, analysis of variance was used. Categorical variables were compared using either Fisher's exact test or the Pearson χ^2^ test when comparing across all 3 de-escalation groups. A multivariable analysis by logistic regression was performed for predictors of non-bleeding-related de-escalation. Variables were included in the model based on potential association with de-escalation or if the *P* value by univariate analysis was < 0.1. *P* values were 2-tailed with a significance level of < 0.05. Analysis was conducted using SPSS version 23 (IBM Canada, Markham, ON).

## Results

### Patient demographics and clinical characteristics

Of the 1854 patients, 209 (11.3%) had de-escalation, and 6 (0.32%) had a lateral change of P2Y12 inhibitor therapy within 1 year of index PCI. Of the patients being de-escalated, all were on ticagrelor at the time of de-escalation. With respect to the timing of de-escalation, 60.3% of de-escalations occurred prior to discharge following index PCI; the remaining 39.7% occurred among outpatients.

**Figure 1:**
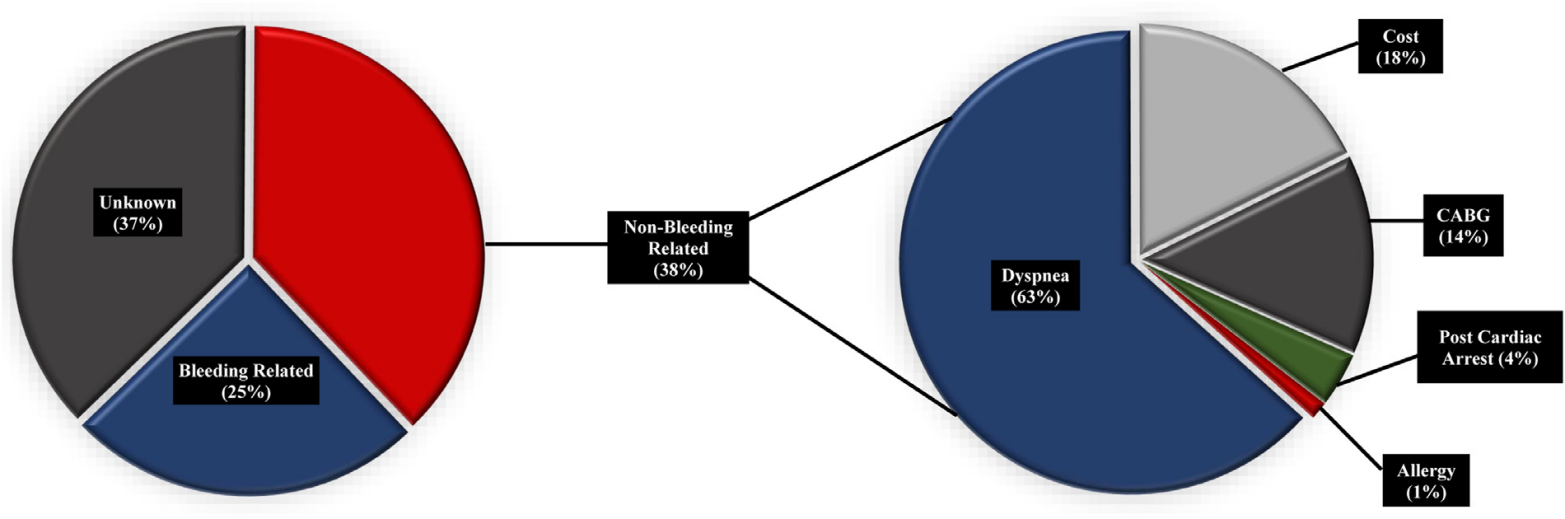
De-escalation of P2Y12 inhibitor, by rationale. Patients with non-bleeding-related de-escalation were further classified by rationale for de-escalation. Coronary artery bypass graft (CABG) indicates CABG conducted after percutaneous coronary intervention.

The average age of those with de-escalation was 65.7 ± 13.1 years, with 89.5% undergoing PCI for ACS indications. Of the de-escalations, 52 (24.9%) were considered bleeding-related, 79 (37.8%) were non-bleeding-related, and 78 (37.3%) had an unknown reason for de-escalation (Fig. 1). Baseline demographics for all patients with de-escalation are shown in Table 1. There were more smokers among the non-bleeding-related and unknown reason groups (*P* = 0.049). In the bleeding-related de-escalation group, there were more patients with atrial fibrillation (*P* < 0.001), on oral anticoagulants (*P* < 0.001), and who underwent PCI for non-ACS indications (*P* < 0.001).

**Table 1:** Baseline characteristics (n = 209)

Characteristic	All (N = 209)	Bleeding- related (n = 52)	Non–bleeding related (n = 79)	Unknown (n = 78)	*P*
Age, y	65.7 (±13.1)	71.0 (±11.5)	63.8 (±13.2)	64.2 (±13.3)	0.101
Female gender	64 (30.6)	19 (36.5)	25 (31.6)	20 (25.6)	0.405
BMI, kg/m^2^	28.6 (±5.8)	28.6 (±5.5)	28.9 (±6.9)	28.4 (±4.7)	0.243
Hypertension	123 (58.9)	32 (61.5)	45 (56.9)	46 (59)	0.873
Dyslipidemia	83 (39.7)	26 (50)	27 (34.2)	30 (38.5)	0.186
Diabetes mellitus	42 (20.1)	13 (25)	14 (17.7)	15 (19.2)	0.579
Diet/lifestyle	13 (6.2)	5 (9.6)	3 (3.8)	5 (6.4)	0.401
Oral medications	20 (9.6)	6 (11.5)	7 (8.7)	7 (9.0)	0.856
Insulin	13 (6.2)	2 (3.8)	6 (7.6)	5 (10.3)	0.683
Smoking history	99 (47.4)	17 (32.7)	42 (53.2)	40 (51.3)	0.049
Current	75 (35.9)	14 (26.9)	31 (39.2)	30 (38.5)	0.297
Former	24 (11.5)	3 (5.8)	11 (13.9)	10 (12.8)	0.321
CAD	50 (23.9)	16 (30.8)	19 (24.1)	15 (19.2)	0.319
Previous MI	41 (19.6)	10 (19.2)	18 (22.8)	13 (16.7)	0.626
Previous PCI	25 (11.9)	6 (11.5)	12 (15.2)	7 (9.0)	0.484
Previous CABG	15 (7.2)	5 (9.6)	3 (3.8)	7 (9.0)	0.333
Family history of CAD	25 (11.9)	5 (9.6)	12 (15.2)	8 (10.3)	0.530
Atrial fibrillation	14 (6.7)	12 (23.1)	1 (1.3)	1 (1.3)	< 0.001
PVD	8 (3.8)	3 (5.8)	3 (3.8)	2 (2.6)	0.647
CHF	19 (9.1)	6 (10.5)	7 (8.9)	6 (7.7)	0.753
Baseline OAC	13 (6.2)	10 (19.2)	0 (0)	3 (3.8)	< 0.001
Indication for PCI					
ACS	187 (89.5)	35 (67.3)	74 (93.7)	78 (100)	< 0.001
STEMI	123 (58.9)	26 (50.0)	49 (62.1)	48 (61.5)	0.326
NSTEMI	53 (25.3)	8 (15.4)	19 (24.1)	19 (24.1)	0.066
UA	11 (5.3)	1 (1.9)	6 (7.6)	4 (5.1)	0.363
Other	22 (10.5)	17 (32.7)	5 (6.3)	0 (0)	< 0.001

Values are mean ± standard deviation, or n (%). Statistical analysis for comparison of continuous variables across all 3 de-escalation groups was performed using analysis of variance. Statistical analysis of categorical variables to allow comparison across all 3 de-escalation groups was performed using a Pearson's χ^2^ test. OACs are warfarin, dabigatran, rivaroxaban, and apixaban. ACS (STEMI, NSTEMI, and UA), indication for PCI: Other; stable CAD, staged PCI, ROSC, and heart failure.

ACS, acute coronary syndrome; BMI, body mass index; CABG, coronary artery bypass graft; CAD, coronary artery disease; CHF, congestive heart failure; MI, myocardial infarction; NSTEMI, non-ST elevation MI; OAC, oral anticoagulant; PCI, percutaneous coronary intervention; PVD, peripheral vascular disease; ROSC, return of spontaneous circulation; STEMI, ST-segment elevation MI; UA, unstable angina.

### Reasons for de-escalation

In those that underwent bleeding-related de-escalation, rationales were as follows: 28 (53.8%) needed concurrent anticoagulation; 14 (26.9%) had active bleeding or were felt to be at increased bleeding risk; and 10 (19.2%) had undergone PCI for non-ACS indications. Rationales for non-bleeding-related de-escalation included the following: for 50 (63.3%), dyspnea from ticagrelor; for 14 (17.7%), the cost of the first-line P2Y12 agents; for 11 (13.9%), they had undergone coronary artery bypass graft surgery following PCI and were not put back on ticagrelor or prasugrel; for 3 (3.8%), post–cardiac arrest reasons; and for 1 (1.3%), allergy (Fig. 1). Of the patients who were de-escalated due to dyspnea from ticagrelor, none (0%) had absolute contraindications to prasugrel, and 12 (24%) had the de-escalation prior to discharge from index hospitalization. In those that were de-escalated due to the cost of first-line P2Y12 Inhibitors, 8 (57.1%) were de-escalated prior to discharge from index hospitalization.

### Outcomes

**Figure 2:**
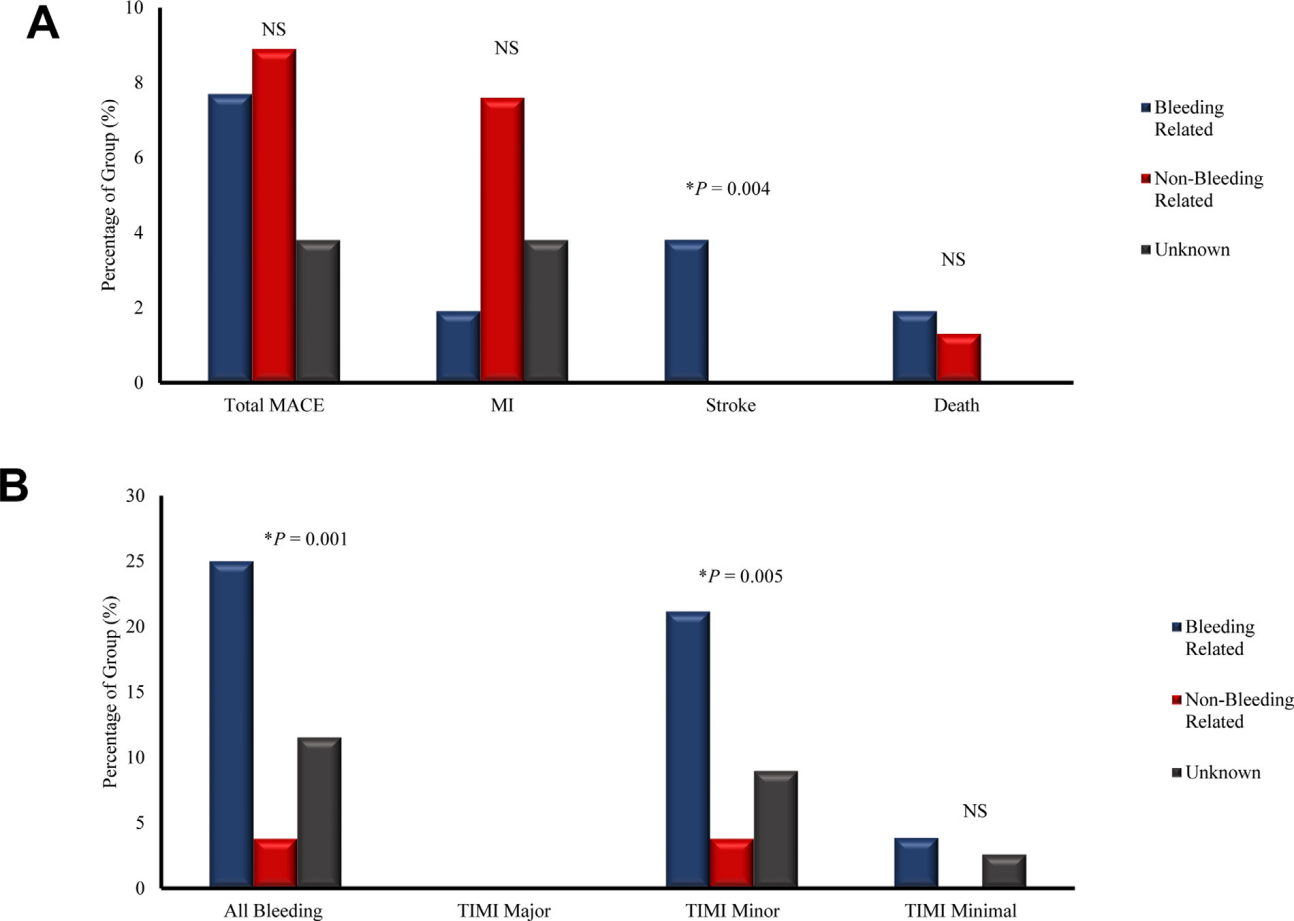
(**A**) The primary outcome of major cardiovascular events (MACE) is shown. The primary outcome is represented as a percentage of events per de-escalation group and separated by the individual components of MACE. (**B**) The safety outcome is represented as a percentage of events per de-escalation group and separated by thrombolysis in myocardial infarction (TIMI)—major, minor, and minimal. Bleeding-related de-escalation is shown in **blue**; non-bleeding-related de-escalation is shown in **red**; and unknown rationale for de-escalation is shown in **black**. Statistical analysis was performed using Pearson's χ^2^ test to allow for comparison across all 3 de-escalation groups. **Asterisk** indicates statistical significance. NS, nonsignificant.

Within 1 year of index PCI, the primary outcome occurred in 14 (6.7%) patients, with 50% of instances occurring in the non-ideal de-escalation group. Of the MACE in the de-escalation cohort, 50% occurred in the non-bleeding-related de-escalation group. MACE occurred in 7 of the 79 (8.8%) in the non-bleeding-related rationale group, 4 of the 52 (7.7%) in the bleeding-related rationale group, and 3 of the 78 (3.8%) patients in the unknown rationale group (*P* = 0.43; Fig. 2). Hospitalization for myocardial infarction occurred in 7.6% of patients who underwent non-bleeding-related de-escalation, 1.9 % of patients who underwent bleeding-related de-escalation, and 3.8 % of patients for whom reasons for de-escalation were unknown (*P* = 0.29). Death occurred in 2 patients—1 in the bleeding-related de-escalation group and 1 in the non-bleeding-related de-escalation group (*P* = 0.51). Ischemic stroke occurred in 2 patients, both of whom underwent bleeding-related de-escalation (*P* = 0.04; Fig. 2).

The safety outcome of bleeding occurred in 25 (11.9%) of the 209 patients. TIMI bleeding occurred in 25%, 3.8%, and 11.5% of patients in the bleeding-related rationale, non-bleeding-related rationale, and unknown rationale groups, respectively (*P* = 0.001). Among the 13 patients who underwent bleeding-related de-escalation, with bleeding events, 8 (61.5 %) were originally de-escalated for bleeding while on a potent antiplatelet medication or because of being identified as being at high risk of bleeding. For the bleeding events, no patients experienced TIMI major bleeding, 21 (10.0%) had TIMI minor bleeding, and 4 (1.9%) had TIMI minimal bleeding. TIMI minor bleeding occurred in 21.2%, 3.8%, and 8.9% of patients in the bleeding-related rationale, non-bleeding-related rationale, and unknown rationale groups, respectively (*P* = 0.005). TIMI minimal bleeding occurred in 3.8%, 0.0%, and 2.6% of patients in the bleeding-related rationale, non-bleeding-related rationale, and unknown rationale groups, respectively (*P* = 0.25).

**Figure 3:**
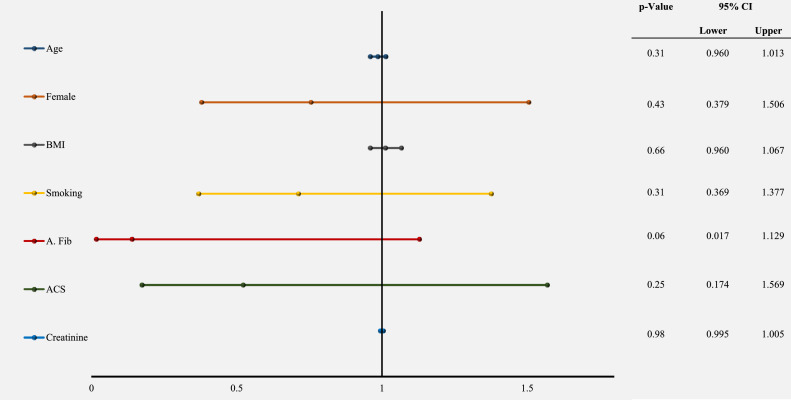
Results of multivariable analysis for predictors of non-bleeding-related de-escalation are shown. Forest plot (**left**) and table (**right**) of a multivariable analysis by logistic regression using age in years (**dark blue**), female gender (**orange**), body mass index (BMI; kg/m^2^; **grey**), smoking history (**yellow**), baseline atrial fibrillation (A. Fib; **red**), acute coronary syndrome (ACS) as the indication for percutaneous coronary intervention (**green**), and creatinine (μmol/L; **light blue**), as predictors of non-bleeding-related de-escalation. CI, confidence interval.

A multivariable analysis was performed with the following covariates: age, body mass index, female gender, smoking history, baseline atrial fibrillation, ACS as the indication for PCI, and creatinine, for predictors of non-bleeding-related de-escalation. Baseline oral anticoagulant use and non-ACS indications for PCI were excluded from the model due to co-linearity. The logistic regression analysis showed no significant predictors for non-bleeding-related de-escalation (Fig. 3).

## Discussion

Our study evaluated the current practice of de-escalation in a contemporary “real-world” cohort and demonstrated several significant findings: (i) a large percentage (11.3%) of patients undergoing PCI had de-escalation of antiplatelet therapy, with almost 60% of the de-escalations occurring prior to hospital discharge; (ii) among patients undergoing de-escalation, the majority had either non-bleeding-related de-escalation or no clearly documented rationale; (iii) among patients with non-bleeding-related de-escalation, the rationale for switching to clopidogrel was often reasons that could have been addressed with the use of prasugrel to avoid the switch and prevent exposure to ischemic risks; and (iv) there was a numerical trend for increased myocardial infarction among patients with non-bleeding-related de-escalation.

De-escalation has been observed previously in 5.3%-13.6% of patients prior to discharge from index hospitalization.^1^^,^^7^^,^^8^ Consistent with this finding, 11.3% of patients in our cohort underwent de-escalation, with many de-escalations occurring prior to discharge after an ACS and index PCI. In prior studies, common reasons for de-escalation included active or high risk for bleeding, need for concurrent anticoagulation or intolerance of side effects, and adverse drug events.^1^ Carrabba et al. evaluated the “appropriate” use of ticagrelor or prasugrel in patients presenting with ACS and found that escalation in P2Y12 agents was attributed to high on-treatment platelet reactivity while on clopidogrel.^10^ No long-term assessments were made in the study that could be used to draw conclusions on de-escalation. Unique in our larger study is an assessment of the rationale for de-escalation. We observed a trend for atrial fibrillation to be associated with bleeding-related de-escalation, likely due to the need for concurrent anticoagulation. We did not find any significant associations of atrial fibrillation with non-bleeding-related de-escalation. Notably, most of the de-escalations were either non-bleeding-related or occurred for unknown reasons, potentially putting patients at-risk of ischemic complications, especially as the de-escalation occurred early after PCI.

Ticagrelor and prasugrel have both been shown to be superior to clopidogrel in preventing ischemic outcomes among patients with ACS who undergo PCI.^2^^,^^3^ The size of our study precluded definitive conclusions on the true impact of ischemic outcomes. Confirmation with largerprospective studies may further reinforce this concept.

Intractable dyspnea is a frequent side effect of ticagrelor and not uncommonly limits the use of this medication. This was the most common reason for non-bleeding-related de-escalation in our cohort. However, in the absence of absolute contraindications to prasugrel, ticagrelor dyspnea should not preclude the use of first-line P2Y12 inhibitors. Furthermore, in our study, 24% of patients de-escalated for ticagrelor-related dyspnea that occurred prior to discharge during index hospitalization. As this side effect frequently occurs shortly following the introduction of ticagrelor and not uncommonly subsides over time, de-escalation potentially could have been avoided with the continued use of this medication. As none of the patients who were de-escalated due to ticagrelor dyspnea had absolute contraindications to prasugrel while it was available, de-escalation could have been avoided in this entire cohort with a lateral change to the alternative first-line P2Y12 inhibitor. A second common explanation for non-bleeding-related de-escalation is that patients underwent cardiac surgery and subsequently were not reinitiated on their original potent P2Y12 inhibitor following the procedure. Similarly, coronary artery bypass graft surgery should not prevent the used of guideline-directed antiplatelet therapy. In fact, evidence supports the benefits of these agents among ACS patients who undergo coronary artery bypass graft surgery.^11^^,^^12^ In an attempt to avoid cardiac surgery as a perceived barrier, increased education or prescription support tools may be considered in the future to improve adherence to guideline-supported therapies.^13^ Finally, cost of the brand-name potent P2Y12 inhibitors was a third common reason for non-bleeding-related de-escalation, which was the case for 17.7% of patients, with 57.1% of these de-escalations occurring prior to discharge from index hospitalization. Although financial restrictions can be a barrier to guideline-directed antiplatelet prescribing, de-escalating this cohort places patients on suboptimal therapy, according to the best evidence. Generic prasugrel has been approved recently by Health Canada, and generic ticagrelor's application is currently under review.^14^^,^^15^ Although the introduction of potent generic P2Y12 inhibitors will not completely eliminate medication cost, and even the most rudimentary of post-ACS medication regimes can remain a financial barrier to many Canadians, generics do come with a substantial cost savings and in part assist patients who lack financial coverage, thus enabling patients to come closer to receiving best evidence-based therapy.

Our study did observe a statistically significant increased rate of TIMI bleeding among patients who underwent bleeding-related de-escalation. Most of these TIMI events (61.5%) occurred in patients whose indication for de-escalation was an elevated risk of bleeding or active bleeding while on a potent P2Y12 inhibitor. These data support the guideline-based rationale that although de-escalation should be avoided when possible, it is sometimes justifiable to balance ischemic benefits with risk of bleeding.^1^

Recently, brand name prasugrel was discontinued in Canada by its distributor. Previous Canada-based studies^16^^,^^17^ have demonstrated very low rates of prasugrel prescription as the initial P2Y12 inhibitor following ACS and PCI, ranging between 0.4% and 12.3%. In contrast, clopidogrel use in the same studies ranged from 63.6% to 65.5%, demonstrating that a second-line medication universally is more commonly prescribed than the guideline-recommended first-line P2Y12 inhibitors. Consistent with this finding, our cohort had an exceptionally low prasugrel prescription rate of 0.32%, in comparison to a clopidogrel prescription rate of 54.9%. The lack of patients on prasugrel as an initial therapy, the absence of prasugrel use for a lateral switch in therapy, the absence of absolute contraindications to prasugrel in the ticagrelor dyspnea cohort, and the relatively disproportionate prescription of clopidogrel all support our hypothesis that the drug has been underutilized and thus has potential for increased prescription. **I**ntracoronary **S**tenting and **A**ntithrombotic **R**egimen: **R**apid **E**arly **A**ction for **C**oronary **T**reatment (ISAR-REACT) 5, the largest randomized study of prasugrel vs ticagrelor among patients with ACS, showed a reduction in death, myocardial infarction, and stroke with prasugrel in the absence of increased bleeding.^9^ This finding is reflected in the most recent guidelines, which suggest that prasugrel be considered in preference to ticagrelor for patients with ACS undergoing PCI.^18^ Given the timing of discontinuation in Canada, physician prescribing patterns did not have the opportunity to reflect the findings in ISAR-REACT 5 that prasugrel is superior. Companies may be willing to consider this finding an impetus to reintroduce prasugrel to the Canadian market. As brand-name prasugrel has now been discontinued in Canada, restricting the market to a single first-line P2Y12 inhibitor, we fear that patients are being offered inferior therapy and deprived of the benefits of a first-line evidence-based agent. Generics are coming to Canada; thus, the findings from our study should provide context for a more evidence-based approach to the use of P2Y12 inhibitors.

**Figure 4:**
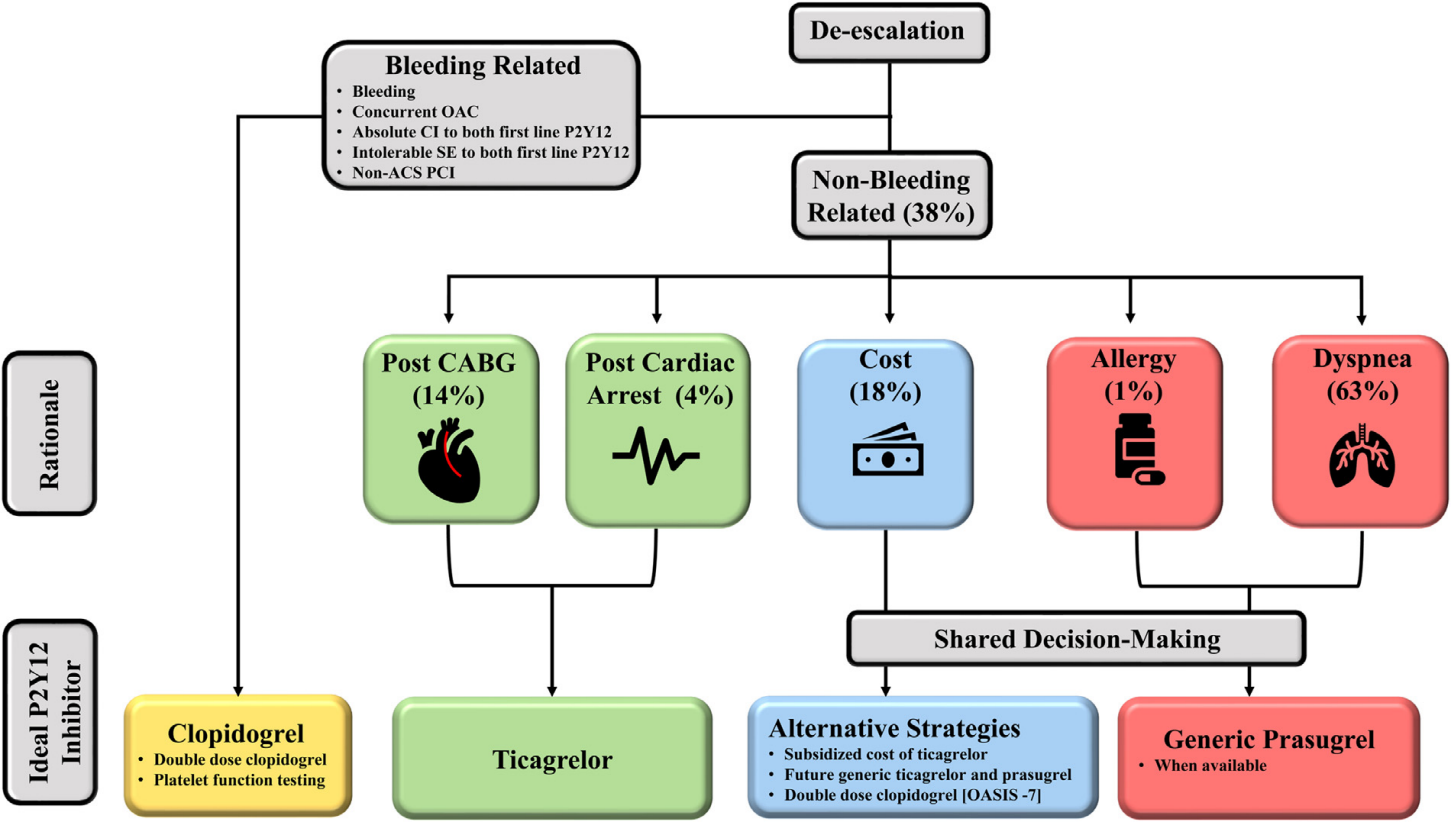
Indication for de-escalation and recommended ideal P2Y12 inhibitor. ACS, acute coronary syndrome; CABG, coronary artery bypass graft; CI, contraindication; OAC, oral anticoagulant; OASIS, **O**rganization to **A**ssess **S**trategies in **I**schemic **S**yndromes; PCI, percutaneous coronary intervention; SE, side effects.

With the current market being restricted to a single first-line agent, physicians need to evaluate the risk to patients and avoid de-escalation when possible. While we await arrival of generics, physicians can consider several alternatives to avoid de-escalation, including the following: (i) use of government- and company-subsidized programs to decrease cost for patients; (ii) early cessation of acetylsalicylic acid in favor of ticagrelor monotherapy, for those with increased bleeding risk^19^; (iii) doubling of clopidogrel in the early post-ACS period for patients for whom de-escalation is unavoidable^20^; and (iv) consideration of platelet function testing to evaluate an individual's risk for de-escalation^21^^,^^22^ (Fig. 4). Ultimately, the use of antiplatelet therapy, particularly when de-escalation is indicated and alternative strategies are being considered, should be a process of shared decision-making. This will account for both physician and patient preference, balancing the bleeding risk with ischemic benefit to tailor therapy for a particular individual.

## Study Limitations

There are some limitations to our study. First, this was a single-centre study, and therefore our data may not reflect practice patterns at other centres across the country. However, prior Canada-based studies have documented similar underutilization of prasugrel, relative to the evidence in support of the drug as a first-line agent.^16^^,^^17^ A unique aspect of our study was the ability to delve into physician justification with respect to the change of antiplatelet therapy; thus, our study provides context and a possible explanation of practice patterns that include underutilization of the drug. Second, in a proportion of patients, we were unable to identify a rationale for de-escalation. This limitation reflects the retrospective nature of our study and means that both the rate of non-bleeding-related de-escalation and associated adverse outcomes may have been underestimated. In our review of patient charts, we endeavoured to determine the rationale for de-escalation even if it was not explicitly articulated. Notably, this cohort was reported in our analysis, as it highlights an additional “real-world” barrier to optimal antiplatelet prescribing—namely, that antiplatelet medications are not uncommonly changed by physicians other than the discharging prescriber. Third, a proportion of patients underwent PCI for non-ACS indications and initially were placed on a potent P2Y12 inhibitor. These patients were subsequently identified by clinicians and de-escalated to clopidogrel accordingly. It was our goal to gain a comprehensive understanding of the condition of all patients undergoing P2Y12 inhibitor de-escalation, thus allowing us to reflect on the optimal use of anti-platelet therapy. We feel that this group of patients reflect real-life practice, and so we have included this cohort in our analysis. However, we acknowledge that evidence to support use of potent antiplatelets in non-ACS PCI is lacking and that inclusion of this cohort in our analysis may limit its generalizability. Finally, our methodology did not enable an understanding of physician decision-making at the time of de-escalation; thus, it is possible that circumstances we were not aware of may have justified the switch in therapy.

## Conclusion

De-escalation of P2Y12 inhibitor therapy is common, with a substantial proportion of patients undergoing non-bleeding-related de-escalation. We have demonstrated that prasugrel has been underutilized, relative to its support from the evidence base and guidelines. With the use of prasugrel and generic ticagrelor when available, 81% of non-bleeding-related incidences of de-escalation in our study could have been avoided. Given the potential risk of ischemic complications, strategies should be considered to encourage both the upfront use of potent P2Y12 inhibitors and alternative strategies to non-bleeding-related de-escalation.
